# Interaction between stimulus contrast and pre-saccadic crowding

**DOI:** 10.1098/rsos.160559

**Published:** 2017-02-01

**Authors:** Mehmet N. Agaoglu, Susana T. L. Chung

**Affiliations:** School of Optometry, University of California, Berkeley, 360 Minor Hall, Berkeley, CA 94720-2020, USA

**Keywords:** saccadic eye movements, crowding, perisaccadic perception, spatial vision

## Abstract

Objects that are briefly flashed around the time of saccades are mislocalized. Previously, robust interactions between saccadic perceptual distortions and stimulus contrast have been reported. It is also known that crowding depends on the contrast of the target and flankers. Here, we investigated how stimulus contrast and crowding interact with pre-saccadic perception. We asked observers to report the orientation of a tilted Gabor presented in the periphery, with or without four flanking vertically oriented Gabors. Observers performed the task either following a saccade or while maintaining fixation. Contrasts of the target and flankers were independently set to either high or low, with equal probability. In both the fixation and saccade conditions, the flanked conditions resulted in worse discrimination performance—the crowding effect. In the unflanked saccade trials, performance significantly decreased with target-to-saccade onset for low-contrast targets but not for high-contrast targets. In the presence of flankers, impending saccades reduced performance only for low-contrast, but not for high-contrast flankers. Interestingly, average performance in the fixation and saccade conditions was mostly similar in all contrast conditions. Moreover, the magnitude of crowding was influenced by saccades only when the target had high contrast and the flankers had low contrasts. Overall, our results are consistent with modulation of perisaccadic spatial localization by contrast and saccadic suppression, but at odds with a recent report of pre-saccadic release of crowding.

## 1. Introduction

The size and distribution of photoreceptors are not uniform across the human retina; in particular, the size of the cone photoreceptors is the smallest and the density of cone photoreceptors is the highest at the fovea [1]. This ‘foveated’ structure necessitates quick realignment of the higher-resolution part of the retina with the objects of interest in the environment (i.e. saccadic eye movements) for taking full advantage of its higher acuity. In fact, humans make several saccades per second. In addition to the lower acuity of peripheral vision, crowding—the deleterious effect of visual clutter on object recognition [2–5], also imposes a fundamental limit on spatial vision, especially in the periphery [6,7]. Directing gaze toward a crowded object reduces or completely eliminates crowding. Crowding affects saccade-landing errors [8].

Saccadic eye movements have other profound consequences on information processing in the visual system. Objects that are briefly flashed around the time of saccades are grossly mislocalized[9–13]. In addition, there is also a profound loss of visual sensitivity around the time of saccades, often referred to as saccadic suppression [14,15]. Strong facilitatory effects of impending saccades (on recognition of isolated targets) at the future retinal locations of attended targets have also been demonstrated [16]. Extensive research has been conducted on which stimulus parameters modulate perisaccadic mislocalization, saccadic suppression and associations/dissociations between perisaccadic localization and recognition [11–13,17–27]. More specifically, brief flashes in an otherwise dark environment are perceptually shifted in the same direction as the saccade [11–13]. In the presence of visible visual references, perceptual distortions take a form which can best be described as a compression of visual space—perceptual shifts toward the saccade target [9,18,28]. Moreover, the strength of saccadic compression depends on the stimulus contrast and the adaptation state of the observer [19,26]. Low-contrast stimuli are compressed more than high-contrast ones [19,20,22]. Near-threshold stimuli (which depend on the adaptation state) are also compressed more than suprathreshold stimuli. Saccadic suppression is also more effective on low-visibility stimuli [17].

Crowding is modulated by the contrast of the target and flankers [29], and depends strongly on target--flanker similarity [6,30–33]. The crowding zone, defined as the target--flanker distance, within which object recognition is impaired due to the presence of flankers, scales with eccentricity. Crowding in the normal periphery is not isotropic: radially arranged flankers are more damaging than tangentially arranged ones [34]. These radially elongated crowding zones are similar to the spatial distribution of saccade-landing errors, which also scales with eccentricity. A recent computational model of crowding asserted that saccade-confounded image statistics can explain the aforementioned characteristics of crowding zones [35]. Considering the similarities between oculomotor metrics and crowding zones, and the parallelism between the modulations of perisaccadic distortions and crowding by the same set of stimulus parameters, the primary aim of this study was to examine the relationship between pre-saccadic crowding and stimulus contrast.

There are conflicting reports as to whether impending saccades release crowding of the saccade target [36,37]. Harrison *et al*. [36] used tilted Gabor stimuli to measure orientation discrimination with and without four vertical flanking Gabors during fixation and before impending saccades. They found that making a saccade to the target Gabor improved performance just before saccade onset. Although the authors interpreted this finding as a release of crowding, it is unclear whether crowding strength itself was reduced before saccades because they did not measure unflanked performance *before saccades*. Although these authors did measure the unflanked performance *during fixation*, using this measurement as the baseline might be misleading because pre-saccadic performance improvements might be due to compulsory shifts of attention to saccade targets, and hence, it may affect crowded and isolated targets similarly. Moreover, the Gabor stimuli were followed (Experiment 1) and preceded (Experiments 1 and 2) by dynamic noise patches at the same locations in Harrison *et al*. [36]. As proposed by van Koningsbruggen & Buonocore [38] (see also [39]), this effect might be due to ‘saccadic unmasking’ where the effect of a forward or backward mask is reduced before saccadic eye movements [40,41]. Recently, we used letter stimuli and directly tested the effect of backward masks in addition to flankers on pre-saccadic letter recognition [37]. We found pre-saccadic improvements in performance when the target letter was crowded *and* masked; however, as similar improvements were also present when the target letter was only masked (but not crowded), crowding strength itself remained unchanged before saccades compared to fixation. In addition, we found no evidence for any improvement in letter recognition when the target letter was only crowded (but not masked) with the same temporal onset. The discrepancy between these studies might be due to the stimulus choices. The overall size, eccentricity and separation of the stimuli were comparable between the two studies. However, one used Gabors and the other used letters. Letters might invoke relatively higher-level processes, and might obstruct any potential change in performance. Therefore, the secondary aim of our study was to test whether or not saccadic uncrowding can be obtained with Gabor stimuli in the absence of masks.

## 2. Material and methods

### 2.1. Participants

Seven observers with normal or corrected-to-normal vision (20/20 or better in each eye) participated in the study. One of the observers was the first author (MNA), and the remaining ones were naive as to the purpose of the study.

### 2.2. Apparatus

Visual stimuli were presented on a 32 inch Display++ display (Cambridge Research Systems, UK) at a resolution of 1920 × 1080 pixels, and a frame rate of 120 Hz. Participants sat at a distance of 76 cm from the display centre with their head stabilized with a chin/head rest. Eye movements were recorded monocularly (right eye) at 1000 Hz with an Eyelink 1000 (SR Research) infrared video-based eye tracker. At the beginning of each block of trials, we performed a calibration using the standard nine-point fixation procedure. All visual stimuli were generated in Matlab (R2012b) (MathWorks, MA) by means of the Psychophysics Toolbox 3 [42,43], and its Eyelink extensions [44]. Observers responded via a keyboard.

### 2.3. Stimuli and procedures

**Figure 1.**
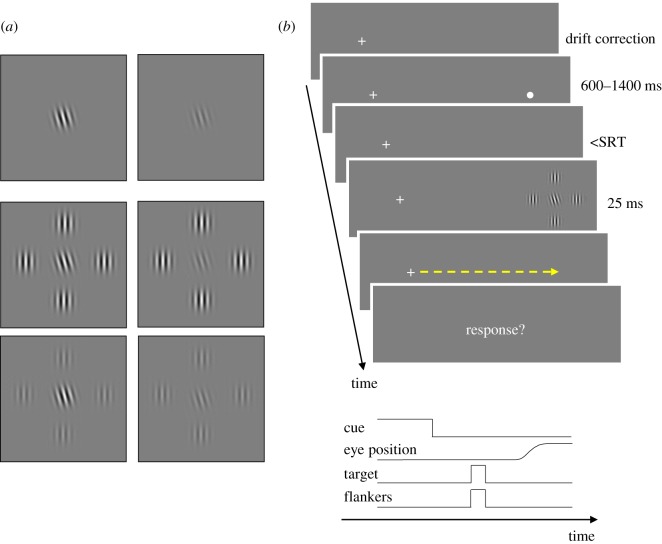
Stimuli and procedures. (*a*) The target Gabor was presented either alone or with four flanking vertical Gabors. The contrast of the target and flanker Gabors could be either high or low, resulting in six different combinations. (*b*) Each trial started with correcting small offsets in eye tracking (i.e. drift correction) while observers fixated at the centre of the display. For the sake of clarity, mostly the right side of the display is shown in the figure, and neither the display nor the Gabor stimuli are drawn to scale. A small placeholder dot indicated the location of an upcoming target. The offset of the placeholder signalled observers to make a saccade toward this location in the saccade blocks. In the fixation blocks, however, they simply ignored the offset of this placeholder and remained fixated at the centre of the display. After a variable delay that was adjusted for each observer individually, the Gabor stimuli were presented. (*c*) The time course of events in a given trial. The timings were set such that observers made a saccade after the stimulus presentation in the majority of trials.

The task of the observers was to report whether a peripherally (at 15 deg eccentricity in the right visual field) presented target Gabor was tilted clockwise or counter-clockwise. The target Gabor could be presented alone (unflanked condition), or flanked by four vertically oriented Gabors. All Gabor stimuli had a spatial frequency of 2 cpd, with the standard deviation of the Gaussian envelope set to 0.5 deg (1 period). There were six different spatial configurations of the stimuli that were randomly interleaved within a block of trials. A high-contrast (100% Michelson contrast) or low-contrast (25% for six observers, 50% for one observer^1^) target Gabor could be presented in isolation, or could be flanked by four high-contrast or low-contrast vertical Gabors. Figure 1*a* illustrates each stimulus configuration. The target--flanker separation (centre-to-centre) was 4 deg, a separation that was sufficient to cause significant crowding at a testing eccentricity of 15 deg according to ‘Bouma's law’ for traditional spatial crowding [5,29,45]. In different blocks, observers were asked to either maintain fixation at the central cross at all times, or make a saccadic eye movement toward a peripheral placeholder as soon as it disappeared. Fixation and saccade blocks were randomly interleaved; however, in order to get sufficient data for different time bins before saccade onset, many more saccade blocks were run. More specifically, each observer ran 600 trials and 3000 trials in the fixation and saccade conditions, respectively.

In both the fixation and saccade conditions, each trial started with a ‘drift correction’ where offsets in the gaze position across trials (possibly due to head movements) were removed. A small placeholder (a small white dot of 0.27 deg in diameter) was presented at 15 deg to the right of the fixation cross, and indicated the location of the target Gabor. The vertical location of the target was randomly jittered by +1, 0 or −1 deg from the horizontal meridian so that observers could not preprogramme their eye movements to the same location without paying attention. The placeholder stayed on the display for a random duration uniformly sampled from 600 to 1400 ms. In the saccade blocks, the offset of the placeholder prompted the observers to make a saccadic eye movement as soon as possible to the remembered location of the placeholder. After a brief interval, the Gabor stimuli were presented for three frames (approx. 25 ms). The duration of the blank frame between the offset of the placeholder and the onset of the Gabor stimuli was adjusted using a method similar to Hunt & Cavanagh [46] and Harrison *et al*. [36]. Briefly, median saccadic reaction time (SRT) was computed for a block of trials, and 50, 100 or 150 ms were subtracted from this value to tailor this variable delay for the next saccade block. For fixation blocks, we used delays from the immediate previous saccade block to closely match stimulus timings in the two conditions. Finally, observers gave their responses after a 750 ms delay using the left or right arrow keys on a keyboard, with an unlimited response time. The procedure is summarized in figure 1*b*. There were 50 or 100 trials within a block of trials. Each observer ran one or two block(s) of practice trials to familiarize himself/herself with the equipment and the experimental task. Practice trials were excluded from further analyses.

In a separate procedure before the main experiment, the amount of tilt required for 80% orientation discrimination was obtained for each observer. In this tilt experiment, all stimuli and procedures were identical to those described above with the following exceptions: (i) only the target Gabor was presented (no flankers), (ii) observers performed the task only during steady fixation at the centre of the display, (iii) the target tilt from vertical was varied from 0° to 15°, with at least six different tilt amounts to accurately fit a psychometric function and (iv) there was no vertical jitter in the position of the target Gabor. Trials for low-contrast and high-contrast Gabors were randomly interleaved within a block of trials. In total, there were at least 12 stimulus conditions (2 contrast × 6 tilt amount), and observers completed at least 30 trials per condition (360 trials) at their first visit. At each following visit, observers completed at least 20 more trials per condition (240 trials), and the data from the most recent two visits were used to obtain 80% threshold tilts for the main experiment.

### 2.4. Data analysis

All analyses were performed offline. Saccades were detected using a velocity criterion of 30 deg s^−1^ and an acceleration threshold of 8000 deg s^−2^. Trials were excluded from analysis if any of the following events occurred: (i) the gaze during fixation drifted outside a 2 × 2 deg^2^ virtual square; (ii) a saccade was made within 300 ms of target presentation in the fixation condition; (iii) no saccade was made in the saccade condition; (iv) the Gabor stimuli were presented earlier than 200 ms, or later than 25 ms before saccade onset; (v) observers blinked during stimulus presentation; (vi) saccades landed outside a 4 deg window around the location of the placeholder; (vii) saccade duration in the saccade condition was shorter than 20 ms or longer than 80 ms.

#### 2.4.1. Equal bin width and conventional statistics

We computed the discrimination performance in the saccade condition of the main experiment as follows. Trials were first sorted in an ascending order of time-to-saccade onset (TSO), i.e. the temporal distance between the stimulus and saccade onsets. A moving window with a width of 40 ms was used to pool data in different time bins with increments of 10 ms from −150 ms to −30 ms TSO (negative signs mean timing before the saccade onset). Trials falling into a given time bin were bootstrapped to estimate the mean and the standard error of the performance for each observer separately. Bootstrapping was done by randomly sampling the trials in a certain bin with replacement. We repeated this procedure 1000 times for each time bin. We computed performance in the fixation conditions with a similar approach. We performed pre-planned paired *t*-tests to compare the performance at a certain time bin and the performance in a corresponding fixation condition. We also repeated the bootstrapping procedure by pooling data from all observers, and comparing performance in the fixation and saccade conditions by using bootstrapped 95% confidence intervals. This approach produced a pattern of results similar, if not identical, to those reported in the remainder of this text. In order to determine the effect of stimulus configurations, we performed a repeated measures ANOVA for the fixation and saccade conditions separately. To investigate the effect of TSO on pre-saccadic judgements, we performed one-way repeated measures ANOVA for each spatial configuration of the stimuli. Whenever the data failed to pass the Shapiro–Wilk normality test, we performed the Friedman repeated measures ANOVA on ranks. For these statistical test, we used SigmaPlot (v11.0) software package (Systat Software Inc., Erkrath, Germany).

We converted each per cent correct value to a *z*-score to compute the crowding strength. Crowding strength was defined as the difference in *z*-scores between the unflanked and flanked stimulus conditions. This is an important point because per cent correct is not a linear metric of performance. For instance, the amount of stimulus contrast change needed to improve performance in a two-alternative forced choice task by 10% is different when the initial performance is at chance (50%) versus when it is at 80%. The same statistical tests were performed as those listed above to determine the effect of TSO and stimulus configuration on crowding strength.

#### 2.4.2. Frequency binning and permutation analysis

Computing observers' performance based on time bins with equal widths has one assumption. The number of trials within each bin more or less should be similar so that performance estimation at different bins has similar statistical power. Despite our efforts to uniformly sample the TSO between 150 ms and 25 ms before saccade onset, the distribution of the number of trials in all conditions revealed a non-uniform sampling (figure 5). Therefore, we repeated all analyses with an alternative method, frequency binning. In short, for all stimulus conditions we specified a fixed number of bins for TSO (*n* = 4), and adjusted the centre and the width of each bin such that the number of trials in each bin was similar. As a consequence, each data point within a condition had equal statistical power.

One disadvantage of frequency binning is that this procedure results in numerically different bin parameters (i.e. slightly different bin centres and bin widths) for each observer, rendering conventional statistical tests (ANOVA, *t*-test, etc.) across observers inapplicable. Therefore, we performed a permutation analysis over data pooled across all observers. As all observers completed an equal number of trials, the contribution of each observer on the average performance is similar. In order to test whether or not TSO had a significant effect on performance or crowding strength, we simulated the predicted performance from the null hypothesis that saccades do not have any effect on performance or crowding strength. To do so, we assigned a random TSO to each and every saccade trial and performed the frequency binning. We bootstrapped this shuffling of trials 10 000 times to obtain 95% confidence intervals for the prediction of the null hypothesis. We then performed frequency binning on the actual data and computed the discrimination performance and crowding strength. Performance values which fell outside the 95% confidence intervals of the null hypothesis were interpreted as evidence against the null hypothesis. In other words, points outside the confidence intervals mean that saccades do have a significant effect on performance.

## 3. Results

**Figure 2.**
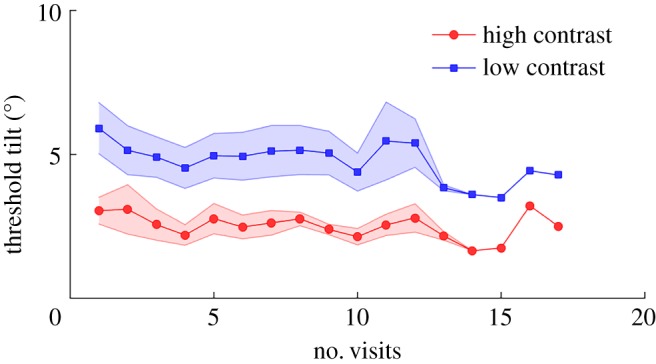
Threshold tilts corresponding to 80% discrimination performance across visits. Shaded regions represent ± s.e.m. across observers (*N* = 7). Tilt thresholds remained fairly similar across visits. Note the ‘conjugate’ (i.e. coupled) fluctuations in high- and low-contrast conditions.

Before the main experiment, we measured the amount of tilt required for each observer to reach at least 80% correct orientation discrimination in the unflanked fixation conditions (for both low- and high-contrast Gabors). At every visit for the main experiment, we repeated this measurement to overcome the potential effects of perceptual learning over time because the main experiment required thousands of trials and perceptual learning has been shown to reduce crowding and its extent [47,48]. Another reason for adjusting the tilt of the target Gabor at each visit was to compensate for potential day-to-day fluctuations in visual sensitivity; as observers' visits were not always at the same time of the day, phases of their circadian rhythm and previous physical activity could have influenced their performance. Overall, we found no discernable effect of number of days (visits) on thresholds (figure 2). However, it is worth noting the coupled changes in thresholds in the two contrast conditions.

**Figure 3.**
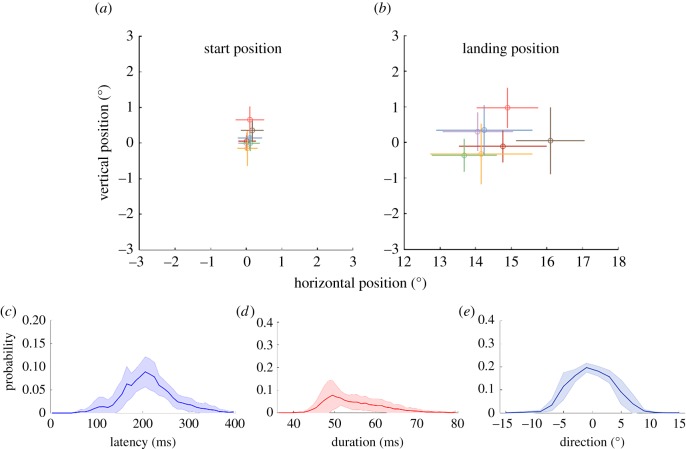
Various eye movement metrics in the main experiment. Individual average starting (*a*) and landing (*b*) position of saccades. Error bars represent s.d. of position. Bottom row shows the distributions of saccade latency (*c*), duration (*d*) and direction (*e*) averaged across observers. Shaded regions represent s.e.m. obtained from bootstrapping (resampling by replacement with 1000 repetitions). In panel (*e*), saccades made along the horizontal meridian correspond to 0^o^ direction. Different colours represent different observers. Saccade landing positions were adjusted for the trial-to-trial variability in vertical position. Owing to this vertical jittering, the distribution of saccade directions is rather broad.

We adopted a stringent set of criteria to include trials for analyses (see Material and methods). On average, 60% (±5%) of trials were included in the following analyses. Interestingly, when we included all trials which satisfied only the temporal criterion for the saccade trials (that the Gabor stimuli should be presented between 200 ms and 25 ms before saccade onset) in the analyses, the results were qualitatively similar and did not affect the main findings of the study. Figure 3 summarizes various saccade metrics for the trials that were included in the analyses.

**Figure 4.**
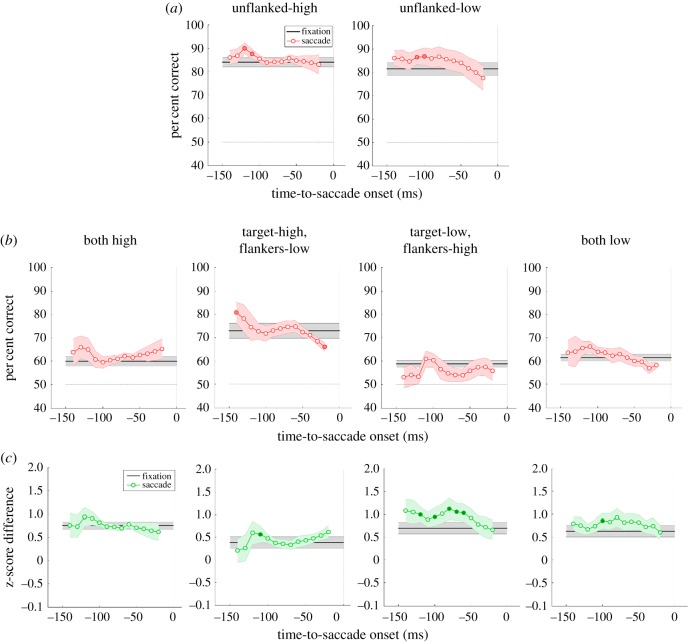
Results of the main experiment. (*a*) Percentage of correct responses in the baseline (unflanked), and (*b*) flanked conditions. Each panel plots tilt discrimination performance as a function of TSO (0 ms representing the saccade onset). Black horizontal lines and red lines represent performance in the fixation and saccade conditions, respectively. (*c*) Crowding strength in terms of *z*-score differences as a function of TSO. Grey lines represent fixation conditions whereas green lines represent saccade conditions. In all panels, filled symbols represent significant difference from fixation performance. Shaded regions represent s.e.m.

Figure 4 summarizes all behavioural data. In the fixation conditions (black horizontal lines in figure 4), as expected, performance significantly dropped in all flanked conditions compared to the unflanked baseline conditions (paired *t*-tests, *p* < 0.027), the well-known crowding effect. A paired *t*-test confirmed that there is no significant difference between the two unflanked conditions (high versus low contrast: *t*_6_ = 0.940, *p* = 0.383). A one-way repeated measures ANOVA on flanked performance revealed a highly significant main effect of contrast combination between the target and the flankers (*F*_3,18_ = 10.986, *p* < 0.001). Bonferroni-corrected *post hoc* multiple comparisons showed that although performance in the target-high flankers-low condition was significantly better than the other three flanked conditions, there was no other pairwise difference among flanked conditions.

Crowding strength can be quantified by computing the difference between performance in the flanked and the corresponding (i.e. the same contrast) unflanked conditions. Figure 4*c* shows crowding strength in terms of *z*-score difference instead of per cent correct difference because per cent correct is not a linear metric of performance. A one-way repeated measures ANOVA revealed a significant main effect of contrast combination of crowding strength (*F*_3,18_ = 3.684, *p* = 0.031). Bonferroni-corrected *post hoc* comparisons showed only one significant difference: the two conditions where the target had high contrast (target-high flankers-high versus target-high flankers-low, *t*_6_ = 3.104, *p* = 0.037). Crowding is known to be affected by target--flanker similarity (e.g. [30]). The more similar they are, the stronger the crowding. This is most apparent in the conditions where the target had high contrast. Crowding was stronger when the flankers also had high contrast than when they had low contrast [29]. However, when the target had low contrast, the effect of similarity was virtually absent; the performance in the target-low flankers-high condition, where one would expect weak crowding due to dissimilarity, was statistically indistinguishable from that in the target-low flankers-low condition (*p* > 0.05). We think that increased lateral masking or surround suppression by high contrast flankers might have cancelled out the potential reduction in crowding due to dissimilarity, thereby resulting in this statistical equality.

In the saccade conditions, discrimination performance showed fluctuations based on TSO. A two-way repeated measures ANOVA on performance in the saccade conditions revealed a main effect of contrast (*F*_5,30_ = 29.366, *p* < 0.001) and TSO (*F*_12,72_ = 2.248, *p* = 0.018). The interaction between contrast and TSO was not significant (*F*_60,360_ = 1.108, *p* = 0.283). We further performed a separate one-way ANOVA for each saccade condition with TSO as the main factor. This analysis showed a significant effect of TSO in the unflanked target-low condition (*F*_12,72_ = 2.833, *p* = 0.003) but no effect in the unflanked target-high condition (*F*_12,72_ = 1.373, *p* = 0.199). In the flanked conditions, we found a significant main effect of TSO only in the target-high flankers-low condition (*F*_12,72_ = 2.377, *p* = 0.012). Similar analyses revealed no significant effect of TSO on crowding strength in all conditions (figure 4*c*).

We further sought to determine the exact timing at which performance (and crowding strength) in the saccade conditions differed significantly from that in the fixation conditions. In the unflanked baseline conditions (red lines in figure 4*a*), performance was significantly higher in the saccade condition compared with the fixation only between −130 ms and −110 ms TSOs (filled symbols in figure 4*a*). In the flanked conditions (figure 4*b*), only in the target-high flankers-low condition were there significant differences between saccade and fixation conditions; performance in the saccade trials started higher than that in the fixation condition 150 ms before saccade onset, and declined as the stimuli were presented closer to saccade onset. Although the overall performance was higher (both high-contrast condition) or lower (target-low flankers-high) in some other conditions, none of these differences reached significance. However, there was a decreasing trend in performance with TSO in the both-low contrast condition, albeit it also failed to reach significance.

Comparison of crowding strength in the fixation and saccade conditions revealed that impending saccadic eye movements never released crowding or reduced it below fixation levels (figure 4*c*). In some cases, the intention to make a saccade led to even stronger crowding (e.g. the middle two panels in the bottom row of figure 4).

**Figure 5.**
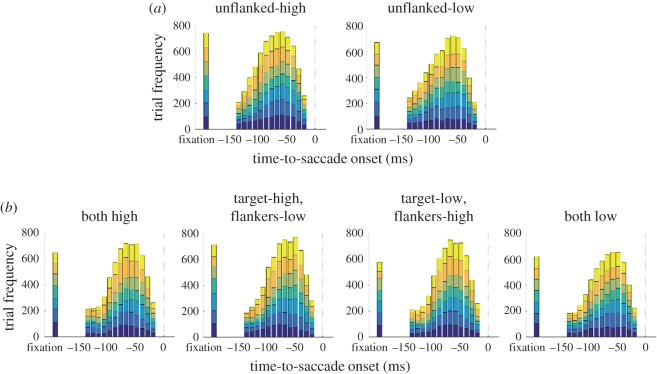
Distribution of the number of trials across time and conditions. (*a*) Unflanked conditions. (*b*) Flanked condition. Different colours in the stacked histograms represent different observers.

We have presented the results based on classical binning (i.e. with equal bin widths) thus far. This method works best when each bin has fairly similar number of trials, otherwise estimates across different time bins will have varying statistical power in resolving an effect. During the main experiment, we manipulated the delay between saccade cue and target display such that in the majority of trials, the Gabor stimuli were presented before saccade onset. Overall, our presentation method proved successful. In figure 5, we plotted the distribution of the number of trials in each condition and time bin. Although, as desired, we obtained many more trials before saccade onset than after, this method resulted in rather non-uniform distribution of trials across time. Therefore, the question arises as to whether the behavioural results reported so far suffered from varying statistical powers across different bins.

**Figure 6.**
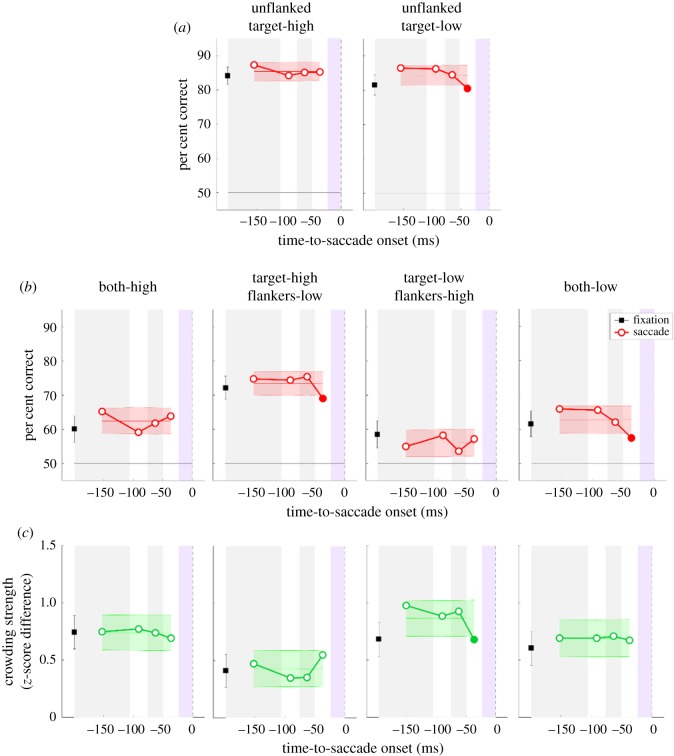
Results based on frequency-binning and permutation analysis. Plots (*a,b*) show discrimination performance in the unflanked and flanked conditions, respectively. Plot (*c*) shows crowding strength in terms of *z*-score difference. Black square symbols represent data from the fixation conditions, whereas red circles represent data from the saccade conditions. Red (*a,b*) and green (*c*) shaded regions represent the mean and 95% confidence intervals predicted from the null hypothesis (i.e. no effect of TSO). Grey and white successive shaded regions represent different time bins. Purple shaded regions represent the time interval excluded from analyses. Filled circles represent significant difference from null hypothesis in the saccade conditions.

To this end, we re-analysed the data in the saccade conditions with frequency binning (i.e. with unequal bin widths). In addition, frequency-binning results in roughly similar but numerically different bin parameters for each observer, which precludes the usage of conventional statistical tests (such as ANOVA and *t*-test). Therefore, we also performed a permutation analysis to determine the effect of impending saccades (i.e. TSO) on performance and crowding strength (see Material and methods for details). Figure 6 summarizes the results for all conditions (the data from fixation conditions remained unaffected and, therefore, are re-plotted from figure 4). First, as before, we did not find any effect of TSO on performance in the unflanked target-high condition whereas there was a decreasing trend in the unflanked target-low condition, which eventually reached significance in the last time bin (figure 6*a*). Similarly, in the flanked conditions where flankers had high contrast (the first and third panels from left in figure 6*b*), there was no effect of impending saccades on performance. However, confirming our results using ordinary binning, we found that performance worsened as the stimuli were presented closer to the saccade onset in the flanked conditions where the flankers had low contrast. Interestingly, even though there was a decreasing trend in performance in the both-low contrast condition, it did not reach significance with a more conventional ANOVA test whereas it was highly significant in the frequency binning coupled with permutation analysis (the rightmost panel in figure 6*b*).

Crowding strength in none of the conditions (except one) showed any effect of TSO. In the target-low flankers-high condition, there was a decreasing trend, which eventually reached significance less than 50 ms before saccade onset. Overall, two analysis methods produced similar results, and resulted in statistically identical conclusions.

## 4. Discussion

We investigated the relationship between pre-saccadic orientation discrimination with and without flankers and compared it to performance in the absence of eye movements. In the fixation conditions, our results suggest that crowding can be modulated by the absolute and relative contrasts of the target and flanker, replicating previous accounts [29,31,49–52]. Previous research showed that increasing the target contrast alleviates crowding whereas reducing it results in stronger crowding [29,49,50]. Stronger crowding when the flankers have higher contrast than the target compared with when they all have the same contrast cannot be explained by target--flanker similarity because it predicts weaker crowding for the former case. Target salience defined as local relative contrast might account for this result [49]. Another possibility is that surround suppression might have dominated over the target–flanker similarity effect when the flankers had higher contrast [51]. Moreover, it has been shown that increasing the contrast of both the target and flankers leads to weaker crowding [29,31,53]. Although our results for dissimilar target and flanker contrasts are consistent with the current literature, similar performance and crowding strength in the both-low and both-high conditions are not (figures 4 and 6, bottom rows). However, this apparent inconsistency can be explained by different tilt amounts used for the low-contrast and high-contrast target conditions in the present study. Here, we intentionally equated the unflanked discrimination performance in the low- and high-contrast conditions by varying the amount of tilt, because our aim was to investigate the effect of contrast on pre-saccadic crowding, not on classical crowding (during fixation). Having similar baseline performance in the low- and high-contrast target conditions allowed us to investigate the potential pre-saccadic changes in performance over a wider range because using the same tilt amount in both contrast conditions would have limited the potential modulation of performance, and might have led to ceiling/floor effects.

In the saccade conditions, we found significant modulations of performance by impending saccades only when the target had low contrast and was unflanked, or when the flankers had low contrast regardless of the target contrast (figures 4 and 6, top and middle panels). In terms of crowding strength, we found a significant decrease only in the target-low flankers-high condition; the rest of the data showed no effect of impending saccades on crowding strength.

The lack of any effect of TSO in the unflanked target-high condition (figure 6, top-left panel) *and* the presence of a decrease in performance with TSO in the unflanked target-low condition are consistent with previous reports. Michels & Lappe [19] investigated the contrast dependency of saccadic suppression and perisaccadic spatial mislocalization. They found larger compression (i.e. mislocalization toward the saccade target) *and* stronger saccadic suppression (i.e. lower detection rate) as stimulus contrast decreased with very similar time courses. The differential effect of saccadic suppression on unflanked orientation discrimination depending on target contrast is obvious here (figure 6*a*). However, it is unclear how a stronger compression would affect performance in the unflanked low-contrast target condition. If the apparent size of the target shrinks due to compression, one would expect a decreasing trend with TSO in performance as we reported here for the low-contrast unflanked case. However, a previous study where a dissociation between the effects of saccades on the ‘what’ and the ‘where’ attributes of objects has been demonstrated, rendering this interpretation very unlikely. Luo *et al*. [54] directly measured the perceived size and location of a horizontal bar briefly flashed around the time of saccades. They found that although the perceived location of the bar was shifted toward the saccade target indicating a compression of perceptual space, the perceived size of the bar was not compressed accordingly. At least two additional studies showed supporting evidence that the perceived size of solid objects centred at the saccade landing point are not affected by saccades [55,56].

Because low-contrast stimuli have been shown to undergo stronger compression [19], one would predict a larger decrease in performance with TSO for low-contrast, than for the high-contrast flankers, as the low-contrast flankers might be perceived as closer to the target Gabor, and that crowding has been reported to depend on the perceived locations of the target and flankers, instead of the physical ones [57,58]. This prediction is validated by our findings; although there was no clear pre-saccadic modulation in performance when the flankers had high contrast, there was a decrease in performance with TSO when they had low contrast. However, as we did not measure the perceived locations of the flankers, this interpretation remains to be a speculation. Nevertheless, our findings with low-contrast flankers are consistent with Matsumiya & Uchikawa's finding that a four-bar stimulus with a small gap between each pair of bars was perceived to be compressed, i.e. the apparent distance between the bars is reduced, although a solid rectangle was not affected by saccades [55]. On the other hand, perisaccadic distortions are either weak or absent for highly visible or high-contrast stimuli [19,20,22]. This is consistent with the lack of pre-saccadic modulation of performance in the both-high and the target-low flankers-high conditions in this study.

### 4.1. Previous work on pre-saccadic uncrowding

Previously, to the best of our knowledge, three studies presented evidence in favour of pre-saccadic uncrowding whereas one did not find any evidence for it [36,37,59,60]. In the following, we discuss in detail the similarities and differences between these studies and the present one to understand the apparent discrepancy among different studies. Table 1 tabulates the stimulus choices and parameters, tasks, observer profiles, and different experimental conditions used in these studies.

**Table 1.** Stimulus choices and parameters in several studies on pre-saccadic crowding.

	Harrison *et al*. [36]	Wolfe & Whitney [59]	Lin *et al*. [60]	Agaoglu *et al*. [37]	This study
stimulus type	target: tilted Gabor (2 cpd)	target: faces	upright faces for both the target and flankers	target: letters (L, H, T, or N)	target: tilted Gabor (2 cpd)
	flankers: 4 vertical Gabors (2 radial, 2 tangential)	flankers: 2 upside down faces (Exp. 1), 4 upside down faces (Exp. 2)		flankers: letters (K, M, R, E, F, Z, I)	flankers: 4 vertical Gabors (2 radial, 2 tangential)
task	orientation discrimination, tilt angle adjusted for 75% correct	face expression discrimination (disgusted versus less disgusted) *and* 5AFC confidence rating	match-to-sample face recognition	letter recognition	orientation discrimination, tilt angle adjusted for 80% correct
size	cropped by a 1 × 1 deg^2^ placeholder	2 × 3 deg^2^	3 × 4.5 deg^2^	1 deg	s.d. of the Gaussian envelope 0.5 deg
eccentricity	7 deg (Exp. 1)	10 deg	12 deg (Exp. 1),	8 deg	15 deg
	7.7 deg (Exp. 2)		approximately 9.5 deg (Exp. 2),		
			12 deg (Exp. 3)		
duration	23.5 ms	200 ms in the fixation, approximately 70 ms in the saccade conditions	30 ms	35 ms	24 ms
contrast	100%	—	—	1.7 (Weber fraction)	50%, 100%
blocked or interleaved	interleaved	blocked	interleaved	blocked	blocked
target--flanker spacing (in terms of target eccentricity)	0.17 (Exp. 1), 0.14, 0.21, 0.29, 0.5, 0.71 (Exp. 2)	0.3 (Exp. 1), 0.3 to 0.5 (fixation), 0.25 to 0.45 (saccade) (Exp. 2)	unflanked (Exp. 1), 0.29 radially, 0.42 tangentially (Exp. 2), 0.3, 0.35, 0.4, 0.55, 0.7 radially (Exp. 3) (tangential spacings not specified)	0.16 (Exp. 1), 0.38 (Exp. 2)	0.27
visual masks	yes	no	no	no for Exp. 1, yes for Exp. 2	no
no. of observers	5	4 (Exp. 1), 2 (Exp. 2)	5	4 (Exp. 1), 3 (Exp. 2)	7
experienced observers	5	4 (Exp. 1), 1 (Exp. 1)	2	1	1
analysis performed on	data pooled across observers	data from each observer separately in Exp. 1, and data pooled across observers in Exp. 2	data pooled across observers	both on individual data and data pooled across observers	both on individual data and data pooled across observers

#### 4.1.1. Harrison *et al*.

Harrison *et al.* [36] investigated pre-saccadic orientation crowding with Gabor stimuli (see table 1 for details) as in this study. The authors reported an increasing trend in performance with TSO when observers made a saccade toward the target, which surpassed the performance in the fixation condition just before saccade onset. The authors concluded that *crowding* of saccade targets is released or eliminated just before saccadic eye movements. The both-high and both-low conditions in this study are most similar to Harrison *et al*.'s because they used same contrast target and flankers. Although we found significant modulations of performance (a decreasing pattern with TSO in the both-low condition), the crowding strength itself in the saccade conditions was virtually the same as in the fixation conditions. In fact, the decreasing performance with TSO in our unflanked low-contrast condition and the both-low condition followed very similar, if not identical, patterns which resulted in no change in crowding strength (figure 6, bottom-right panel). In short, our findings are not consistent with those of Harrison *et al*.

We believe that the apparent discrepancy between our study and Harrison *et al*.'s can be explained by differences in stimuli. The target Gabor in Harrison *et al*.'s study was forward and backward masked (in Experiment 1, and only forward masked in Experiment 2) by dynamic noise masks in addition to being crowded by four vertically oriented flanking Gabors. This stimulus configuration is known to result in ‘super-crowding’ where the combined deleterious effect of the masks and the flankers is larger than the sum of their individual effects [61]. We think that the performance improvement in Harrison *et al*.'s study is largely due to saccadic ‘unmasking’ [38], instead of a release of crowding. The supporting evidence for our claim comes from De Pisapia *et al.* [40] and Fracasso & Melcher [41], who demonstrated that both forward and backward masking can be completely eliminated before saccadic eye movements. Furthermore, Rolfs & Carrasco [62] used backward masking in a pre-saccadic orientation discrimination task and found strong improvements in performance just before saccade onset. These authors also found that the effect was far less pronounced without masks (Rolfs, personal communication).

#### 4.1.2. Wolfe & Whitney

Wolfe & Whitney [59] used face stimuli to measure pre-saccadic crowding (see table 1 for details). They asked observers to report whether the target was face A (with a less disgusted expression) or face B (with a more disgusted expression), a 2AFC task. Observers were also asked to rate their confidence of report on a scale of 1 to 5. The 2AFC task provides an objective measurement of observers' percepts and the confidence-ranking paradigm provides a subjective reading of percepts. The authors combined the responses to both tasks and presented their results based on only this semi-objective semi-subjective metric, on a scale of 1 to 10. They computed receiver operating characteristic curves and took the area under the ROC curve (referred to as AUC from now on) as the performance. The authors found that the AUC was larger in the saccade condition compared with the fixation condition (0.73 and 0.64, respectively). In their Experiment 2, the authors performed a control experiment with two participants (one author and another naive observer) and several target--flanker separations. The authors claimed that any increase in performance with increasing separation would indicate that the effect they reported in Experiment 1 was due to crowding. Although they did measure unflanked performance in both the fixation and saccade conditions in Experiment 2, they did not do so in Experiment 1. Nevertheless, they did not find any difference in performance between the fixation and saccade baseline performance in the control experiment. Interestingly, although they did not find any statistical difference between the fixation and saccade conditions in three out of four target--flanker separations that were used in both conditions, they did report a significantly higher (mean difference = 0.13) AUC in the saccade condition for a single target--flanker separation of 3 deg, which shifted the psychometric function fit to the left of that for the fixation.

We think that the apparent discrepancy between Wolfe & Whitney's findings and ours can also be explained by methodological differences. First of all, as they did not present the results for each task (2AFC and five-level confidence rating) separately, it is difficult to compare their results with ours or other studies mentioned previously. Second, these authors also did not measure unflanked baseline in their main experiment. Third, the control experiment had four flankers although the main experiment had two, and therefore, it is difficult to make a quantitative comparison among experiments. Fourth, in their control experiment where they investigated the spatial extent of crowding, they fitted psychometric functions to performance averaged across two observers. Combined with a significant difference between the saccade and fixation conditions for only one target--flanker separation, the conclusion that the crowding zone or extent is reduced before saccades should be considered with more scrutiny, as also acknowledged by the authors. We think that (i) more and closer target--flanker separations should be tested for both conditions to improve fits, (ii) psychometric function fitting should be carried out on individual data (this point applies also to Harrison *et al*.'s study mentioned previously, especially considering the gross idiosyncratic results from different observers in the crowding literature [63,64]), (iii) corresponding threshold estimates should be averaged across observers and (iv) finally, these estimates should be put through statistical tests for difference between the fixation and saccade conditions. Last but not least, their specific choice of stimulus timings might have led to improved performance in the saccade condition. Because their criterion for turning off the stimuli in the saccade conditions was gaze deviated by more than 0.5 deg from the centre of the display (the number of samples that were part of the saccade varied) *and* because the refresh rate of the display used was 60 Hz, it is quite possible that on average the stimuli were turned off during saccades, at least well into saccades. Given the latency of the Eyelink eye tracker (data transfer delay up to 3.8 ± 0.6 ms depending on filtering options, see Eyelink 1000 functional specifications), the variable number of samples needed to satisfy the position criterion (each sample will add 1 ms), and frame duration of the display (approx. 17 ms), the stimuli would be turned off from less than 1 ms up to approximately 24 ms (e.g. in the case where the gaze moved outside the 0.5 deg limit very close to a vertical retrace of the display and by the time the stimulus software received the sample the vertical retrace was missed, and thus, the software had to wait until the next frame to update the display unless the display was updated asynchronously) into saccades. Assuming that saccade durations are in the range of 30–50 ms for 10 deg saccades [65], this delay in updating the display corresponds to a stimulus eccentricity of significantly smaller than 10 deg. Therefore, the slight difference between the fixation and saccade conditions might be simply a result of timing difference. However, note that this is only a speculation considering that whether or not observers have access to expression information during saccades is not known. All other studies on pre-saccadic crowding, including the present one, included trials where the stimulus was already turned off at least 25 ms before eyes started to move.

#### 4.1.3. Lin *et al*.

Lin *et al*. [60] sought to replicate Harrison *et al*.'s findings with face stimuli to determine whether the assumed saccadic uncrowding mechanism lies before or after where faces are processed in the brain. These authors used upright faces for both the target and flankers, and a match-to-sample task. In Experiment 1, Lin *et al*. examined unflanked face recognition before saccades and during fixation. In Experiment 2, they investigated the crowded face recognition before saccades and during fixation, and finally in Experiment 3, they varied the target--flanker separation to determine the change in crowding extent with impending saccades. The authors reported performance improvements in Experiment 1, which showed a linear increase from 120 ms before saccade onset and peaked at the last time bin (60 to 30 ms) before saccade onset. They also reported a similar increasing trend in Experiment 2 with crowded faces. However, because the largest improvement in Experiment 2 was around 20% where it was around 10% in Experiment 1 (unflanked condition), the authors concluded that crowding is still released. In their Experiment 3, they found this performance increase only for target--flanker separations of 3, 3.5 and 4 deg but not for 5.5 and 7 deg. The psychometric function fit to the fixation was shifted leftward compared with that obtained from the data in the last bin of saccade trials. The authors concluded that crowding is released before saccades and its extent is reduced.

We find this conclusion unwarranted given the inconsistencies across different experiments in Lin *et al*.'s study. First, even though they did find pre-saccadic improvements for unflanked faces, because they chose a different stimulus paradigm for crowded faces, where the eccentricity used was different for each observer, the direct comparison of results from their Experiment 1 and Experiment 2 is not possible. The change in eccentricity of 2.5 deg on average might have led to the slight edge for saccade trials over fixation ones in Experiment 2. Second, even though these authors found an increasing unflanked recognition performance in Experiment 1, they did not factor that in their Experiment 3 (nor did they measure unflanked performance) where they estimated the change in crowding extent, with yet another eccentricity (fixed at 10 deg for all observers).

#### 4.1.4. Agaoglu *et al*.

Admittedly, our results in this study alone cannot prove that saccadic uncrowding reported by other studies mentioned above is not a genuine effect; but that was only the secondary aim of our study. To prove that saccadic uncrowding is not a genuine effect, one needs to directly compare pre-saccadic crowding with and without masks to fully test the effect of the masks, as in a previous study of ours [37]. In that study, we asked observers to report the identity of a target letter in a letter trigram after making a saccade toward the target letter. In Experiment 1, we did not present any mask whereas in Experiment 2 the letters were followed by noise masks. Although we did not find any pre-saccadic improvement in performance when the target and flankers were presented simultaneously in Experiment 1, we found very strong improvements in Experiment 2, when backward masks were used. However, performance improved in both the unflanked baseline and flanked conditions, resulting in no change in crowding strength itself.

#### 4.1.5. This study

In this study, the only condition where we obtained a significant reduction in crowding strength was when the target had low contrast and the flankers had high contrast. One might claim that this finding is due to a floor effect in the flanked condition. In other words, the reduction in crowding strength results from the performance decrease in the unflanked low-contrast condition. This is unlikely as the performance in the target-low flankers-high condition was significantly above chance. Nevertheless, the reduction in crowding was not such that crowding strength became weaker than that in the corresponding fixation condition. The crowding strength was significantly larger than that in the fixation condition beyond 100 ms before saccade onset, and decreased to the same level as in the fixation condition (figure 6, the third panel in the bottom row). In none of the cases explored here, crowding strength before saccades was less than that during fixation. In other words, we did not find any evidence of pre-saccadic uncrowding.

The discrepancy between our current findings and the ones that showed pre-saccadic uncrowding may be due to the choice of stimulus type (Gabors, letters or faces). We think that this is highly unlikely given the absence of such an effect in this study and in Agaoglu *et al*. [37] where we used Gabors and letters, respectively. However, faces might be affected differently by pre-saccadic mechanisms due to their level of processing in the brain. A more controlled study using faces and other complex stimuli where unflanked saccade performance is factored in crowding strength and extent estimates, and ceiling and floor effects are avoided, is needed.

One of the reviewers raised the possibility that differences between studies might be due to the eccentricities used in different studies (table 1). The eccentricities used in the studies where pre-saccadic uncrowding was reported were 7, 7.7 [36], 10 ± 1 [59], 12, 9.5 ± 0.4 and 10 [60] deg whereas in this study and our previous study where we did not find such an effect (due to compensating for improvements in unflanked recognition before saccades), the eccentricities were 15 and 8 [37] deg, respectively. Under normal conditions, human saccades have magnitudes of 15 deg or less [66], so there might be differences in perisaccadic processing for target eccentricities within and beyond this range. However, because all the studies cited above utilized values within this range, and because we showed the absence of pre-saccadic uncrowding for 8 and 15 deg eccentricities, we think that eccentricity is also unlikely to account for differences across studies.

Another comment can be made regarding the extent of crowding. We did not measure the extent of crowding in this study and in our previous study [37], however, Harrison *et al*. and Lin *et al*. did measure it by varying the target--flanker separation. Both groups concluded that pre-saccadic uncrowding depends on the target--flanker separation; pre-saccadic performance showed improvement with TSO only for the closest separations, and this may suggest that the failure to find uncrowding in this study and in our previous study [37] might be simply due to an insufficient range of parameters explored. The target--flanker separations used in different studies are tabulated in table 1. The target--flanker separations for which there was a significant improvement in performance before saccades were 0.29Φ in Harrison *et al*.'s study, and 0.3Φ, 0.35Φ and 0.4Φ in Lin *et al*.'s study (where Φ represents target eccentricity). Note that even though there was an increasing trend in performance for the 0.14Φ and 0.21Φ separations in Harrison *et al*.'s study, this trend turned out to be not significant. Because the ratios between target--flanker separation and eccentricity were more or less similar across studies, we think that differences across studies in target--flanker separations are also unlikely to be the cause of the disagreement of results.

## 5. Concluding remarks

In summary, previous research has shown powerful changes in visibility and drastic mislocalizations before saccades. The primary purpose of this study was to explore the relationship between the absolute and relative contrast of the target and flankers and pre-saccadic changes in visual processing. The secondary aim of our study was to clarify the conflicting reports about saccadic uncrowding with a more primitive stimulus (i.e. Gabors compared to letters or faces). Our results show that pre-saccadic crowding and crowding during fixation were statistically indistinguishable in three out of four combinations of target--flanker contrast. We found a reduction in pre-saccadic crowding only in the condition where the flankers had high contrast and the target had low contrast; however, this release occurred from a much larger crowding strength and ended around crowding levels measured during fixation. Taken together, our results suggest that saccadic uncrowding is not a genuine effect, at least not in the sense that it reduces crowding below the levels measured during fixation. It is likely, however, that our results may not generalize to the rest of the retinal space. It may well be possible to obtain significant changes in crowding strength and its extent at off-target or unattended regions. Nevertheless, we think that saccadic suppression and compression together can account for all results reported here. Further experimentation is needed to quantify the individual contribution of these two processes and test this claim.

## Footnotes

1 For one of the observers, 25% contrast yielded chance-level performance for all tilt amounts, and therefore, we used 50% contrast in the low-contrast condition for this observer.

## Data accessibility

Our data and a script for generating figures can be found in the Dryad Digital Repository: http://dx.doi.org/10.5061/dryad.r9g5c [67].
